# *Drosophila*: A Versatile Model in Biology and Medicine

**DOI:** 10.3390/ijms26115032

**Published:** 2025-05-23

**Authors:** Maria Grazia Giansanti, Roberto Piergentili

**Affiliations:** Istituto di Biologia e Patologia Molecolari (IBPM) del Consiglio Nazionale delle Ricerche (CNR), at Dipartimento di Biologia e Biotecnologie, Università Sapienza di Roma, Piazzale Aldo Moro 5, 00185 Rome, Italy

*Drosophila melanogaster*, the fruit fly, is one of the most famous and widely used model organisms in studying not only general biology problems (such as insect physiology, behavior, evolution and cell biology) but also human conditions. This is thanks to the evolutionary conservation of genes involved in basic cellular functions and to the practical advantages involved, such as low rearing cost, fast life cycle, ample progeny, and advanced formal and molecular genetics tools [1,2]. In addition, research on this organism began at the start of the 20th century [3], making it one of the first and most long-lived models in the history of genetics. Here, we are proud to present our guest-edited Special Issue, “*Drosophila*: A Versatile Model in Biology and Medicine”, showcasing the most recent reports on the use of the fruit fly to investigate the etiopathogenesis of human diseases. In this issue, readers will find ten contributions, which may be accessed here: https://www.mdpi.com/journal/ijms/special_issues/CS6KOD97I2.

Vecchiè and collaborators explored the impact of the angiotensin-converting enzyme inhibitor lisinopril on the metabolic rate of fruit flies. They demonstrated that the drug affects the whole-body metabolic rate in a genotype-dependent manner, supporting existing evidence of a connection between ACEI (angiotensin-converting enzyme inhibitor) drugs and metabolic rate, and offering new insights into this relationship [4].

Steinmetz and collaborators explored the impact of dNox and dSod3 in the egg hardening process and egg laying on the reproductive function of *Drosophila melanogaster*. They concluded that the localization of the ROS-producing enzyme dNox within fruit fly ovaries demonstrates that dNox-derived ROS are involved in the chorion hardening process. They also showed that reduced dSod3 activity impacts egg-laying behavior but not the hardening process. Notably, the localization of these two enzymes largely corresponds to that of their mammalian orthologues, NOX5 and EC-SOD [5].

Elovsson and collaborators built a novel *Drosophila* model of Alzheimer’s disease to study Aβ proteotoxicity in the digestive tract; the model expresses Aβ peptides with a built-in apoptotic sensor. They found that expressing different variants of Aβ1–42 resulted in proteotoxic phenotypes such as reduced longevity, aggregate deposition, and the presence of apoptotic cells [6].

Chen and collaborators showed a potential role for the *mtt* gene in retinal disease using the fruit fly as a model. They presented a congenital stationary night blindness (CSNB) model of *Drosophila* using RNA interference to silence this gene (homologous to the mammalian *GRM6* gene) in fly eyes, underscoring the crucial involvement of *mtt* in fly retinal function, and providing a framework for understanding the pathogenic mechanisms of CSNB [7].

Cheng and collaborators analyzed how the tyrosine metabolism pathway is downregulated in dopaminergic neurons by *LRRK2* overexpression in *Drosophila*. In particular, the authors explore the role of *LRRK2* gene mutations, the leading cause of familial Parkinson’s disease (PD) and a significant risk factor for idiopathic PD cases. Their findings suggest that the downregulation of tyrosine metabolism occurs before pronounced dopaminergic neuron loss [8].

Atienzar and collaborators reviewed the current knowledge on nucleotide repeat expansion diseases (NREDs) and discussed how the fruit fly became a central tool in their study. NREDs are a group of genetic diseases characterized by the abnormal expansion of DNA repeats, and include Huntington’s disease (HD), several spinocerebellar ataxias (SCAs), and myotonic dystrophies type 1 and 2 (DM1/DM2); the authors showed how *Drosophila* models contributed to a better understanding of their clinical and etiological features [9].

Ehrhardt and collaborators propose the fruit fly as an alternative model to higher organisms for in vivo lung research, presenting and discussing its use for investigating airway diseases such as COPD (chronic obstructive pulmonary disease) and asthma, complementing and/or replacing models in higher organisms, thanks to its limited lifespan, evolutionarily conserved signaling pathways and tissue-specific gene modification [10].

Volonté and collaborators analyzed the state of the art in the use of *Drosophila* to investigate histamine function. Histamine has been demonstrated to be a pleiotropic master molecule in pharmacology and immunology, with increasingly recognized roles in the nervous system as well. In their work, the authors summarized the exploitation of *Drosophila* to extend the scientific debate to academia, industry, and the general public regarding the pathophysiological consequences of mutations on genes involved in histamine metabolism and signaling [11].

Vedelek and collaborators showed how *Drosophila* may be used to analyze mitochondrial differentiation during spermatogenesis. In this review, the authors elucidate the dynamic alterations and functions of mitochondria in germ cell development during the spermatogenesis of *Drosophila melanogaster* and highlighted how these studies impact our knowledge about human spermatogenesis in terms of mitochondrial organization and function [12].

Finally, Melnikova and Golovnin summarized the multiple roles of dADD1 and dXNP, *Drosophila* orthologs of the ATRX chromatin remodeler. The authors reported the current state of knowledge about these two proteins in the fruit fly, which are orthologues of the ADD and SNF2 domains of the vertebrate ATRX (alpha-thalassemia mental retardation X-linked) protein, which plays a role in general molecular processes, such as the regulation of chromatin status and gene expression. They also discuss the possible mechanisms of action of complexes involving these proteins [13].

We are grateful to all authors who submitted their work and supported this collection, and to the *International Journal of Molecular Sciences* staff, whose help was invaluable for the success of this editorial project. All articles are open access to readers and distributed under the terms and conditions of the Creative Commons Attribution (CC BY) license 4.0 (https://creativecommons.org/licenses/by/4.0/).
